# Editorial: Anti-MDA5-Positive Dermatomyositis

**DOI:** 10.3389/fmed.2022.941370

**Published:** 2022-05-31

**Authors:** Takahisa Gono, Shuang Ye, Qian Han

**Affiliations:** ^1^Department of Allergy and Rheumatology, Nippon Medical School Graduate School of Medicine, Tokyo, Japan; ^2^Scleroderma/Myositis Center of Excellence, Nippon Medical School Hospital, Tokyo, Japan; ^3^Department of Rheumatology, Renji Hospital, Shanghai Jiaotong University School of Medicine, Shanghai, China; ^4^State Key Laboratory of Respiratory Disease, National Clinical Center for Respiratory Disease, Guangzhou Institute of Respiratory Health, The First Affiliated Hospital of Guangzhou Medical University, Guangzhou, China

**Keywords:** dermatomyositis, interstitial lung disease, anti-MDA5, type 1 interferon pathway, immunosuppressive therapy, antifibrotic agent

Anti-melanoma differentiation-associated gene 5 antibody-positive dermatomyositis (anti-MDA5-positive DM) is a unique clinical phenotype of DM characterized by less involvement of the skeletal muscles, vasculopathy-related cutaneous manifestations such as skin ulcers and palmar papules, and a high incidence, potentially fatal, rapidly progressive interstitial lung disease (RP-ILD) (1, 2). The discovery of anti-MDA5 antibody and the development of a convenient measurement procedure for this autoantibody have led physicians to manage anti-MDA5-positive DM patients earlier and more readily before devastating RP-ILD develops (2, 3). Researchers across the world have been encouraged to determine new aspects regarding the etiology, pathogenesis, pathophysiology, variability of clinical features, independent prognostic factors, and therapeutic strategies of this disease (4–9). However, sufficient research has not yet been carried out to achieve satisfactory outcomes for anti-MDA5-positive DM patients. There are still urgent demands and unmet needs for more efficacious and more robust evidence-based treatment for anti-MDA5-positive DM patients.

This Research Topic aims to increase awareness and bring new insights into this rare but significant disease. On this topic, relevant articles have emerged as follows. In terms of pathogenesis, So et al. validated the results of Nishina et al. (5) and showed a seasonal effect on disease onset in anti-MDA5-positive DM. This study showed that fewer anti-MDA5-positive DM patients experienced disease onset from July to September. However, no particular seasonal pattern was observed in the anti-MDA5 antibody-negative myositis patients. RP-ILD occurred more frequently in patients with disease onset from October to December. This finding suggested that certain environmental factors, such as infections, might be involved in the pathomechanism of this disease. Type I interferon (IFN) is important for host defense against viruses through the induction of antiviral effector molecules that are encoded by IFN-stimulated genes (10). Hu et al. reviewed the pathogenesis of anti-MDA5-positive DM, focusing on the role of the type I IFN system. They mentioned that the dysregulation of the type I IFN system could play a role in the pathogenesis of anti-MDA5-positive DM according to accumulating evidence over the past few years. In fact, increased serum IFNα levels were detected in patients with anti-MDA5-positive DM, and the type I IFN gene signature was upregulated in both peripheral blood mononuclear cells and skin tissues in these patients. Of note, in a recent study, Wang et al. demonstrated that immune complexes (ICs) formed by MDA5 and anti-MDA5 antibodies could potently stimulate the production of IFNα *via* TLR7 in an RNA-dependent manner *in vitro*, which might serve as endogenous type I IFN inducers in anti-MDA5-positive DM (11). In parallel, a report from Lyon suggested that monoclonal antibodies from B cells of anti-MDA5-positive DM patients, primed and selected by type I IFN, could subsequently augment IFNγ production (11). Therefore, considering “interferonopathy” as the endotype of anti-MDA5-positive DM, new targets of the IFN system, such as Janus kinase inhibitors, could be new treatment options.

In terms of clinical practice, reports have emerged regarding predictors of prognosis using peripheral blood, imaging such as positron emission tomography (PET) and radiomics with computed tomography (CT), or comprehensively integrated risk models. Lv et al. indicated that patients with a lower average monocyte count or lymphocyte count in the first 2 weeks after admission had a higher 6-month death risk. Cao et al. elucidated the usefulness of 18F-fluorodeoxyglucose PET/CT for the prediction of prognosis in patients with anti-MDA5-positive DM. This study showed that significant correlations between the maximum standardized uptake value (SUVmax) of the spleen and the serum ferritin levels, myositis disease activity assessment score, bone marrow SUVmax, and bilateral lung SUVmax were observed, suggesting that the activation of the reticuloendothelial system is closely involved in poor prognosis. Xu W. et al. demonstrated that the CT-based radiomic score (Rad-score) was associated with 6-month all-cause mortality. They also incorporated significant clinical predictors, including age, the course of DM, arthralgia, forced vital capacity, arterial oxygen/fraction of inspiration oxygen, lactate dehydrogenase, serum ferritin, C-reactive protein, lymphocytes, and maximum dosage of methylprednisolone, into the Rad-score. The final Rad-score plus model was developed to appropriately predict the 6-month mortality. In addition, Ouyang et al. created a matrix prediction model for the 6-month mortality risk. This model was composed of three items, fever, ferritin ≥1,250 μg/L, and positive carcinoembryonic antigen, and classified three risk groups, a high-risk group (all-cause mortality rate, 78%), a moderate-risk group (all-cause mortality rate, 43%), and a low-risk group (all-cause mortality rate, 25%). The predictive modalities mentioned above should be validated in another cohort to confirm the consistent usefulness of the predictive risk models for all anti-MDA5-positive DM patients worldwide or to adjust those models depending on race.

We should also consider numerous complications, such as infections, pneumothorax, and mediastinal emphysema, under treatment with intensified immunosuppressive therapy in anti-MDA5-positive DM patients in daily practice. Xu Z. et al. reported a rare but life-threatening complication. They described a case series of patients with spontaneous intramuscular hemorrhage (SIH) associated with DM and conducted a literature review. The overall mortality was 60.9% in DM patients with SIH. In most of these patients, hemorrhagic events occurred within 6 months of disease onset, predominantly in anti-MDA5-positive DM patients.

Treatments for anti-MDA5-positive DM are largely empirical and have not been fully addressed in this topic collection. Nevertheless, a real-world JAMI cohort study just came out (12). Unfortunately, the data failed to demonstrate the survival benefits of classic initial triple-combination therapy, i.e., high-dose glucocorticoids, cyclophosphamide, and tacrolimus, in different anti-MDA5-positive DM clusters. The effect of antifibrotics on anti-MDA5-positive DM related RP-ILD has not been fully illustrated, only presented in a case report (13) and a retrospective study with a small sample size (14), suggesting that the addition of antifibrotic may play improve the survival in these patients.

Regarding future perspectives, we need to learn more appropriate management as well as clarify the pathophysiology related to treatment targets for anti-MDA5-positive DM patients through numerous, remarkable further advances. We believe that we can develop novel therapeutic strategies and implement personalized medicine based on the risk of mortality using several uniform predictive models across the world, considering the prevention of various life-threatening complications, to improve the quality of life as well as the prognosis of anti-MDA5-positive DM patients.

## Author Contributions

All authors listed have made a substantial, direct, and intellectual contribution to the work and approved it for publication.

## Conflict of Interest

TG received speaking fees from Astellas, Boehringer Ingelheim, Bristol-Myers Squibb, Janssen, MBL, Nippon Shinyaku, and Ono Pharmaceuticals. The remaining authors declare that the research was conducted in the absence of any commercial or financial relationships that could be construed as a potential conflict of interest.

## Publisher's Note

All claims expressed in this article are solely those of the authors and do not necessarily represent those of their affiliated organizations, or those of the publisher, the editors and the reviewers. Any product that may be evaluated in this article, or claim that may be made by its manufacturer, is not guaranteed or endorsed by the publisher.
